# Lipidomics profiling of skin surface lipids in senile pruritus

**DOI:** 10.1186/s12944-020-01347-y

**Published:** 2020-07-16

**Authors:** Xiaolei Ma, Lulu Lu, Zheng Zhao, Mingru Cai, Na Gao, Gangwen Han

**Affiliations:** Department of Dermatology, Peking University International Hospital, Life Park Road No.1, Life Science Park of Zhong Guancun, Changping District, Beijing, 102206 China

**Keywords:** Lipidomics, Senile pruritus, Skin surface lipids, Skin barrier function, Triacylglycerol, Sphingolipids, ceramides

## Abstract

**Background:**

Senile pruritus is common, yet its etiology remains unknown. Aging-associated skin barrier defects and skin surface lipid (SSL) alterations have been postulated to play important roles in its occurrence. In the present study, the lipidomic profiles of SSLs in elderly patients were examined to better understand the potential causes of senile pruritus.

**Methods:**

Transepidermal water loss (TEWL) was evaluated to assess the skin barrier function. The Ameliorated Kawashima Itch Scale score was used to measure the pruritus severity. Liquid chromatography coupled with tandem mass spectrometry (LC-MS/MS) and multivariate data analysis were employed to investigate SSL alterations.

**Results:**

The results showed that senile pruritus patients had higher TEWL values than control subjects (13.13 ± 4.28 versus 6.71 ± 2.45, *p <* 0.01). LC-MS/MS revealed significant differences in the lipidomic profiles and identified 81 species of SSLs that differed between the two groups. Compared with control subjects, senile pruritus patients had increased levels of ceramides (Cers), diacylglycerols, fatty acids, phosphatidylcholines, phosphatidylethanolamines, phytosphingosines, sphingosines, diacylceryl-3-O-carboxyhydroxymethylcholine, diacylglyceryl trimethylhomoserine, and unsaturated free fatty acids, but decreased levels of triacylglycerol. Cer-EOS, Cer-NDS, and Cer-NS were positively correlated with TEWL value (*p* < 0.05). Pruritus severity score was positively correlated with sphingomyelin, Cer-NP, Cer-AS, Cer-NDS, and Cer-NS, but negatively correlated with Cer-BS, Cer-EODS, Cer-EOS, and Cer-AP.

**Conclusions:**

The present study indicated that patients with senile pruritus have impaired skin barrier function and altered SSL composition. Certain SSL species identified in this study may be potential targets for future studies on the pathogenesis of senile pruritus.

**Trial registration:**

Peking University International Hospital (Number: YN2018QN04; date: January 2019).

## Background

Senile pruritus is defined as chronic itching in elderly people with unknown etiology, and is a diagnosis of exclusion after all other causes of pruritus have been ruled out [1]. With the prolonged life expectancy worldwide in the setting of rapid medical advances, ageing-related skin diseases like senile pruritus are becoming increasingly relevant [2]. Pruritus is the most frequently reported dermatological complaint in the elderly population [3]. This severe itching may not be self-limiting, and can lead to sleep disorders and even psychiatric complications such as depression in certain cases that significantly decrease the quality of life [4]. The pathophysiology of senile pruritus remains unclear, although several causes have been hypothesized, including age-related changes in skin, neurological disorders, and abnormal immunological responses [5]. Thus, research on pruritus in the elderly population is urgently needed.

Skin surface lipids (SSLs) are a mixture of sebaceous gland lipids and intercellular lipids located in the outer layer of epidermal cells. SSLs play an important role in maintaining the skin barrier function and help to regulate various aspects of the integumentary system, including cell proliferation, cell apoptosis, immunity, and inflammation [6]. Abnormalities in SSL composition are involved in the pathogenesis of common skin diseases such as atopic dermatitis (AD), acne, psoriasis, and seborrheic dermatitis [7]. Lipidomics is an emerging field of study that involves large-scale comprehensive evaluation of the end-products of lipid metabolism [8]. Liquid chromatography coupled with tandem mass spectrometry (LC-MS/MS) is a new analytical technique employed in lipidomics that allows the identification and quantification of cellular lipid species [9].

Because little is known about the SSL profiles in senile pruritus, the present study employed LC-MS/MS to compare the SSL profiles in senile pruritus patients with those in control subjects without pruritus to investigate the role of SSLs in the pathogenesis of senile pruritus.

## Methods

### Chemicals and reagents

Acetonitrile (ACN), methanol, isopropanol (IPA), and Optima™ LC/MS Grade were obtained from Thermo Fisher Scientific (Waltham, MA, USA). Sebutape was purchased from CuDerm Corporation (Dallas, TX, USA).

### Participants

The study was reviewed and approved by the Ethics Committee of Peking University International Hospital. Informed consent to participate in the study was obtained from all patients (elderly with senile pruritus) and healthy individuals (elderly without pruritus) before enrollment. Individuals aged > 60 years were eligible for inclusion. Forty participants from the Beijing area were enrolled in the study, including 20 patients with senile pruritus and 20 healthy controls. All 20 patients had physician-confirmed diagnosis of senile pruritus and were able to complete the study. The patients underwent a complete examination and checkup to exclude diabetes, liver or kidney dysfunction, malignant tumor, HIV infection, thyroid disorder, anxiety or depression disorder, psoriasis, atopic dermatitis, ichthyosis, scabies, eczema, bullous pemphigoid, and other dermatoses that can cause skin pruritus. The participants had not received any treatments or drugs that could interfere with the study assessment for 6 months, including cardiovascular, antiepileptic, antibiotic, and antipsychotic drugs. The participants did not bathe or apply topical moisturizer for 24 h before the test. The study protocol ensured that the participants were matched for demographic characteristics of sex and age.

### TEWL measurement

Transepidermal water loss (TEWL) measurements were performed at a site 1 cm below the right knee using a portable VapoMeter (TM300; CK, Cologne, Germany). The measurement time was 8–10 s. Briefly, the VapoMeter was maintained under standard ambient conditions in a cool air-conditioned room at temperature 23 °C and humidity 50%. After the detection probe was placed on the target area, three consecutive readings were collected from the same site and averaged for each participant.

### SSL sampling

Before sample collection, participants were instructed to remain under standard ambient conditions (room temperature 23 °C and humidity 50%) in a cool air-conditioned room for 30 min. Sebum was collected from an approximately 4-cm^2^ area at the same site 1 cm below the right knee using Sebutape. Prior to sebum collection, the collection area was wiped with a 5% saline swab and one Sebutape patch was placed on the target site. The Sebutape patch was left in place for 10 min, and then removed to a sterile centrifuge tube using curved forceps. All samples were immediately stored at − 80 °C until further analysis.

### Itch intensity scale for assessment of pruritus severity

Pruritus severity in each patient was evaluated by the Ameliorated Kawashima Itch Scale (Supplementary Table 1) [10], which rated itch severity on a five-point scale (0, 1, 2, 3, 4) in separate diurnal and nocturnal assessments. The pruritus score was calculated by adding the diurnal score to the nocturnal score (range, 0–8).

### Sample preparation

After retrieval of the samples from the − 80 °C freezer, 1.5 mL of chloroform/methanol (1:2 v/v) was added to each tube. The samples with chloroform and methanol were mixed by vortex vibration for 10 min and placed at − 20° for 12 h. Subsequently, 1.4 mL of supernatant was removed and 0.2 mL of chloroform was added to further extract the lipids. Each tube was then added with 1 mL of deionized water and centrifuged at 3000×*g* for 5 min to stratify the solution. After the stratification, the upper layer was the aqueous phase and the lower layer was the organic phase. A low temperature concentrator (Speed Vac SPD131P; Thermo Fisher Scientific) was used to dry the lipid extracts for later analysis. Before mass spectrometry detection, the lyophilized samples were dissolved in 100 μL of methanol/isopropanol (2:1 v/v). Sample mixtures were also prepared as quality control samples for analytical performance prior to analysis by ultra-performance liquid chromatography coupled with quadrupole time-of-flight tandem mass spectrometry (UPLC-QTOFMS).

### LC-MS/MS analysis and identification

Liquid chromatography was performed using LC instrument (Exion LC AD, AB SCIEX, USA: Exion LC AD, AB SCIEX, USA). LC separation was performed using a Phenomenex Kinetex 1.7u EVO C18 column (2.1 × 50 mm, 100A; Agilent, Santa Clara, CA, USA). The column temperature was 40 °C and the sample dosage was 3 μL. The flow rate was 0.5 mL/min. Mobile phase A was 50% water + 50% ACN + 10 mM ammonium formate and mobile phase B was 10% ACN + 90% IPA + 10 mM ammonium formate. The gradient elution conditions with mobile phase A and mobile phase B are shown in Table 1. MS was performed using a Triple TOF 5600+ (AB SCIEX, Concord, Ontario, Canada) and an orthogonal accelerated TOFMS equipped with an electrospray ion source. The ionization mode of positive and negative ions from an electrospray ionization source was used. The data collection method was TOF-MS-IDA-TOF-MS/MS (*n* = 8). The quadrupole scanning range was *m/z* 50–1500. The detailed ion source conditions are shown in Table 2. An independent reference, Lock-mass ion, via the MS-Dial (ver. 3.70; 17 April 2019) was used to ensure mass accuracy during data acquisition.

**Table 1:** Elution programme of liquid chromatography

Time (min)	Flow (mL/min)	A%	B%
0	0.5	90	10
0.5	0.5	90	10
11	0.5	0	100
17	0.5	0	100
17.1	0.5	90	10
20	0.5	90	10

mobile phase A:50% water + 50%ACN (Acetonitrile) + 10 mM ammonium formate

mobile phase B: 10% ACN + 90% IPA (Isopropanol) + 10 mM ammonium formate

**Table 2:** Ion source parameters of LC-MS/MS

Parameters	Value
curtain gas	30 psi
Gas 1	50 psi
Gas 2	60 psi
capillary voltages	5500(+), − 4500 (−) v
source temperature	550 °C
declustering potential	80 v
collision energy	TOF-MS: 10 v
	TOF-MS/MS: 35 ± 15 v

psi: pounds per square inch

The assigned modified metabolite ions were identified by database searches in the MS-Dial Lipidomics MSP database (http://prime.psc.riken.jb/compms/msdial/mail.html). The chromatographic retention behavior was considered to reduce false-positive matches [11].

### Statistical analysis

Using MS-Dial (Ver. 3.70) and MS-Dial Lipidomics MSP database information transferred into the software, the original mass spectrum data were used for processes such as peak identification, peak filtering, and peak alignment. For multiple linear regression analysis, the results eventually generated a two-dimensional data matrix containing lipid name, peak time, lipid classification, mass core ratio (*m/z*), peak area, and original Excel file data. MetaboAnalyst 4.0 (http://www.metaboanalyst.ca/MetaboAnalyst/) was used to normalize the samples with different comparison requirements, and an Excel file called DATA_mart was obtained. A multivariate analysis, comprising partial least-squares discrimination analysis (PLS-DA), was constructed to determine the distributions and identify metabolic differences between the senile pruritus patients and healthy controls using MetaboAnalyst 4.0. The PLS-DA models were cross-validated using a 10-fold method with unit variance scaling. R^2^ evaluated the fitting of the PLS-DA model while Q^2^ evaluated the prediction ability. If the Q^2^ value was negative or extremely low, it meant that the differences between the two groups were not statistically significant. The PLS-DA model eliminated any changes in the X matrix that were not related to the Y matrix. Therefore, only one prediction component was used to distinguish the two classes.

Comparisons of the two groups related to the intensities of integrated regions were carried out using a two-tailed Welch’s *t*-test, which was performed within MetaboAnalyst 4.0. Values of *p* < 0.05 were considered statistically significant.

## Results

### Clinical data of patients and controls

The clinical data of the patients and control subjects enrolled in the study are listed in Table 3. All of the participants were enrolled from January 2019 to March 2019. The 20 patients with senile pruritus comprised 9 males and 11 females, and had a mean age of 69.05 ± 6.38 years. All patients had complained of pruritus for > 6 months. Dermatology examinations showed cutaneous xerosis, decrustation, or excoration in the lower limbs. The 20 control subjects comprised 8 males and 12 females, and had a mean age of 67.4 ± 6.35 years. The two groups did not differ significantly in age (*p* = 0.208) and sex (*p* = 0.584).

**Table 3:** Clinical data of patients and controls

**No. of Patients**	**P1**	**P2**	**P3**	**P4**	**P5**	**P6**	**P7**	**P8**	**P9**	**P10**	**P11**	**P12**	**P13**	**P14**	**P15**	**P16**	**P17**	**P18**	**P19**	**P20**	**Average**	**SD**
Sex	M	M	M	M	F	M	F	M	M	F	F	F	F	F	F	M	M	F	F	F		
Age (y)	60	65	79	71	60	62	76	68	70	70	78	60	64	63	72	69	80	70	69	75	69.05	6.38
TEWL	13.02	10.4	10.29	14.62	11.38	24.66	17.56	9.84	10.55	15.38	7.59	21.46	8.22	10.05	12.76	11.28	13.13	12.89	16.37	11.07	13.13	4.28
Pruritus Score	1	4	6	4	2	3	7	5	2	2	5	3	2	6	3	2	2	8	6	8	4.05	2.19
**No. Of controls**	**C1**	**C2**	**C3**	**C4**	**C5**	**C6**	**C7**	**C8**	**C9**	**C10**	**C11**	**C12**	**C13**	**C14**	**C15**	**C16**	**C17**	**C18**	**C19**	**C20**	**Average**	**SD**
Sex	F	M	F	F	F	F	F	M	M	F	F	F	F	M	F	M	F	M	M	M		
Age (y)	75	61	65	77	61	77	72	64	73	65	65	71	63	79	71	62	61	60	66	60	67.40	6.35
TEWL	7.41	4.83	5.11	5.35	4.71	7–93	10.46	7.83	9.12	10.86	5.34	4.55	5.26	4.76	5.35	4.66	10.12	5.38	4.94	11.43	6.71	2.45
Pruritus Score	0	0	0	0	0	0	0	0	0	0	0	0	0	0	0	0	0	0	0	0	0	

TEWL: Transepidermal water loss; Pruritus Score: Rated by the Ameliorated Kawashima Itch Scale

### Skin barrier function

TEWL is commonly used for assessment of human skin barrier function. An increase in TEWL usually represents damage to the skin barrier [9]. In this study, senile pruritus patients had significantly higher TEWL values than control subjects (13.13 ± 4.28 versus 6.71 ± 2.45, *p <* 0.01; Table 3). These results suggest that senile pruritus is associated with dysfunction of the skin barrier, consistent with the symptom of xerosis commonly observed in senile pruritus [5].

### SSL profiles

**Fig. 1:**
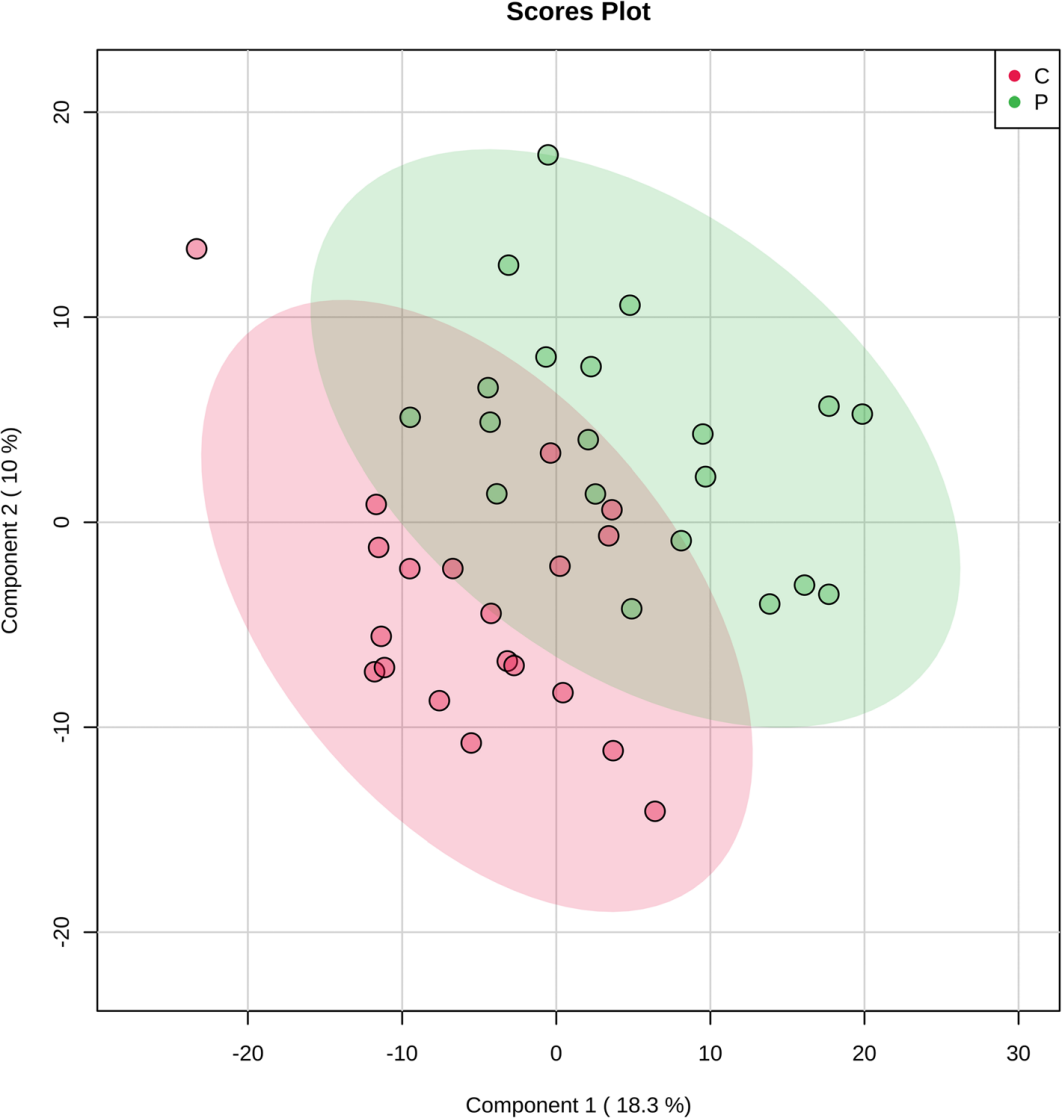
PLS-DA score plot of skin surface lipid (SSL) from senile pruritus patients and healthy person controls. SSL profiles of senile pruritus (green dots) and controls (red dots) are obviously separated. R^2^ = 0.9469, Q^2^ = 0.12092

To understand whether SSL changes occur in senile pruritus, the SSL profiles in the skin of the senile pruritus patients and healthy controls were analyzed using LC-MS/MS. The abundances of all compounds were normalized, followed by peak extraction and molecular characteristic determination of each entity. A PLS-DA, as a supervised multivariate data analysis method, was used to analyze the lipids in the senile pruritus patients versus the healthy controls. Based on the lipid dataset analyses, the SSL profiles in the patient group appeared distinct from those in the control group (R^2^ = 0.9469, Q^2^ = 0.12092; Fig. 1). These results indicate that alterations in SSLs may be related to the development of senile pruritus.

### Differences in SSLs

**Fig. 2:**
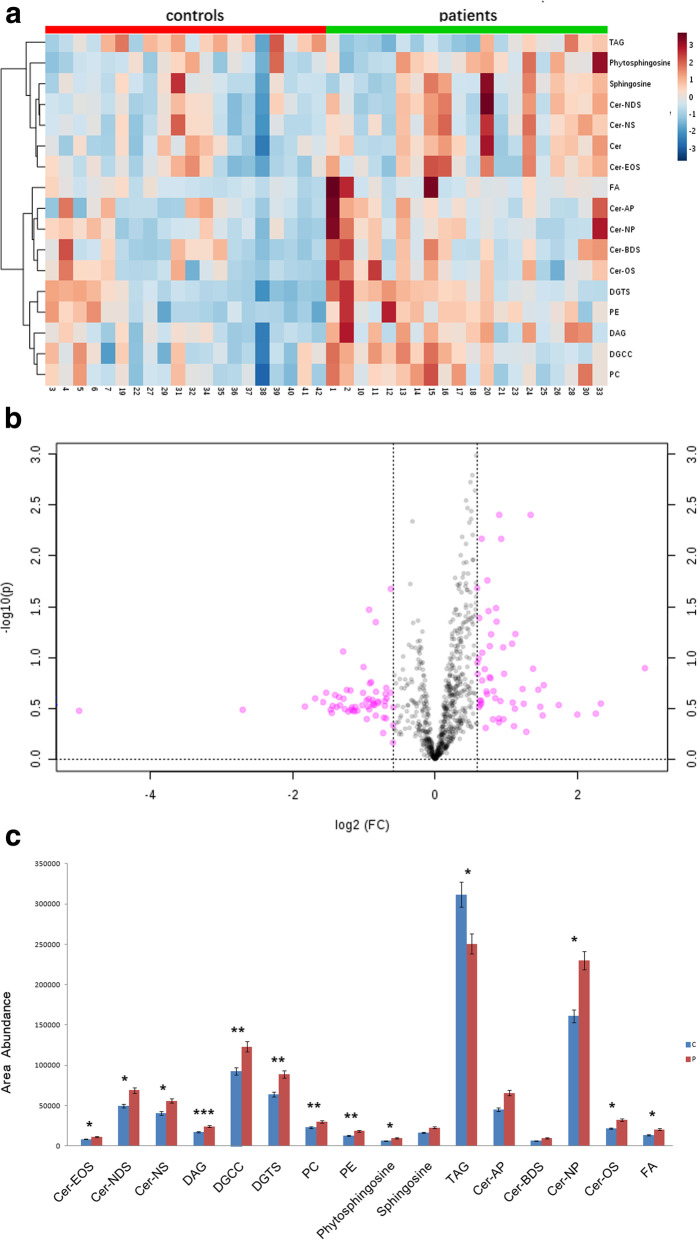
Identification of differential lipids and lipid metabolites between senile pruritus patients and healthy person controls. **a** Heat map of SSL. The color is proportional to the intensity of SSL changes; red color represents upregulation, and blue represents down-regulation. **b** Volcano plot of SSL. The red dot represents 1.2 fold (right) and 0.83 fold (left) of variation and *p* < 0.05. Total 81 lipids with significant change have been identified based on their difference between two groups. **c** The comparison of 16 main class of lipids between idiopathic senile pruritus and controls. Compared to controls, 15 main class of lipids increased and only TAG decreased in senile pruritus. Results showed that there were significantly increased levels of Cer-EOS, Cer-NS, DAG, DGCC, DGTS, PC, PE, Cer-NP, Cer-OS, FA phytosphingsines and decreased level of TAG, while there were no significant changes of the relative amount of Cer-NP, Cer-BDS and sphingosines. *** *P* 0.001, ** *P* < 0.01, * *P* < 0.05

A total of 796 SSLs were detected by LC-MS/MS, and classified into 25 categories. The two groups were subsequently compared for their lipid species. Based on the PLS-DA analysis and Q-value (false discovery rate) evaluation, several parameters were used to identify lipid species with significant differences between the senile pruritus patients and control subjects. These parameters included variable importance for projection > 1, fold change > 1.2 or < 0.83, and Q-value < 0.05. Based on these criteria, a heat map and volcano plot were created (Fig. 2a,b). A total of 81 lipids with significant changes were identified based on their differences between the two groups (Supplementary Table 2). These 81 SSLs belonged to 17 categories according to their biochemical features. The relative amount of each lipid class was calculated and compared between the two groups (Fig. 2c). Several main classes of lipids including diacylglycerols, fatty acids, phosphatidylcholines, phosphatidylethanolaine, phytosphingosine, sphingosines, diacylceryl-3-O-carboxyhydroxymethylcholine, and diacylglyceryl trimethylhomoserine were significantly increased among the SSLs in the senile pruritus patients, while triacylglycerol (TAG) was significantly decreased.

### Associations between SSL alterations and skin barrier damage

To determine whether certain SSL components are related to the impaired skin barrier function in senile pruritus patients, the correlations of TEWL with categories of lipids were analyzed. Among all lipids compared, only several ceramide (Cer) species had positive correlations with TEWL (Table 4). Specifically, levels of Cer-EOS, Cer-NDS, and Cer-NS had significant positive correlations with TEWL value.

**Table 4:** Correlation between TEWL and ceramide levels

Species of Cers	Total Cers	Cer-AP	Cer-BDS	Cer-EOS	Cer-NDS	Cer-NP	Cer-NS	Cer-OS
Correlation Coefficients with TEWL*	0.23336	0.061623	0.21673	0.34001	0.48352	0.08259	0.46858	0.27462
*P* value	0.14	0.7	0.17	0.03	0.002	0.61	0.002	0.08

*Pearson product-moment correlation coefficient was used to show the association between CER profiles and TEWL index. Note that Cer-EOS, Cer-NDS and Cer-NS in the table have significant positive correlations with the corresponding TEWL indexes (*P* < 0.05)

### Correlations of SSL alterations and itch severity in senile pruritus

Severe itching in senile pruritus can significantly affect the quality of life of patients. Therefore, the correlations of itch severity with Cer levels were evaluated. The correlations of SSL alterations with pruritus severity score rated by the Ameliorated Kawashima Itch Scale were evaluated. The comparative results are summarized in Table 5. Generally, sphingomyelin, Cer-NP, Cer-AS, Cer-NDS, and Cer-NS showed positive correlations with pruritus score, while Cer-BS, Cer-EODS, Cer-EOS, and Cer-AP had negative correlations, although statistical significance was not observed. Because sphingolipids were identified as important pruritogenic substances [12], the present results suggest that the significance of sphingomyelin in senile pruritus warrants further investigation.

**Table 5:** Correlation between Pruritus Scores and SSL

Species of SSL	sphingomyelin	Cer-BS	Cer-EODS	Cer-NP	Cer-BDS	Cer-OS	Cer-ADS	Cer-AS	Cer-EOS	Cer-NDS	Cer-NS	Cer-AP
Correlation Coefficients with pruritus scores*	0.24624	−0.02536	−0.03254	0.13395	0.037961	0.11059	0.11935	0.16527	−0.06283	0.14215	0.17252	−0.00483
*P* value	0.13	0.88	0.84	0.41	0.82	0.49	0.46	0.31	0.7	0.38	0.29	0.98

*Pearson product-moment correlation coefficient was used to show the association between SSL and pruritus severity (scaled by pruritus score) in senilepruritus. Note that sphingomyelin, Cer-NP, Cer-AS, Cer-NDS, Cer-NS have positive correlations with pruritus scores, while Cer-BS, Cer-EODS, Cer-EOS, Cer-AP have negative correlations, but *P* value did not reach to statistically significant

## Discussion

The pathophysiology of senile pruritus remains unclear. Cutaneous xerosis is the most common clinical feature in senile pruritus and is considered a consequence of skin aging associated with decreases in sweat and sebum production [3, 13]. Dry skin is also thought to be an important factor for triggering pruritus through alterations in the stratum corneum (SC) barrier function [14]. In humans, SSLs originate from sebum secreted by sebaceous glands and cornified keratinocytes that cover and protect the skin surface and play a role in maintaining skin moisture [15]. As a result, variations in SSL composition can damage the skin barrier function. In turn, skin barrier damage may further alter SSL components and accelerate cutaneous xerosis. Because of the complex interplay among components of the skin barrier, any changes in the skin surface condition, such as SSL composition, may be related to pruritus development. In the present study, LC-MS/MS was used to elucidate the lipid compositions in senile pruritus patients and asymptomatic control subjects.

Epidermal barrier dysfunction is strongly linked to chronic pruritus [16]. Increased TEWL leads to activation of serine proteases such as SC chymotryptic enzymes by increasing the pH on the skin surface [17]. Increased TEWL is also correlated with xerosis [18]. In the present study, senile pruritus patients had higher TEWL values than control subjects, and TEWL value was positively correlated with pruritus severity score (data not shown). Thus, the results indicate an impaired skin barrier function in senile pruritus patients compared with asymptomatic elderly control subjects. Given that AD is an inflammatory skin disease characterized by severe itching and skin barrier damage, it is a good example for understanding the physiological role of the epidermal barrier. When the epidermal barrier function is damaged, external stimulants can enter the skin, cause inflammation, and elicit itch-inducing mediators [19]. Although no significant inflammation exists in senile pruritus, it is reasonable to conjecture that the skin barrier damage in senile pruritus plays a crucial role in inducing a similar itch-scratch cycle to that seen in AD.

Alterations in SSLs have previously been reported in several inflammatory diseases, including AD and acne [15, 20, 21]. In the comparisons of the lipid profiles between senile pruritus patients and healthy controls, the PLS-DA scores showed distinct SSL compositions in the two groups.

Cers are the most abundant lipid constituents among human SSLs, and comprise approximately 50% of the intercellular lipid content by mass. Changes in skin Cer contents are associated with several diseases, including AD and psoriasis [22]. Cer-EOS and Cer-NS are the two main subclasses of Cers. These Cers are related to the formation of the corneocyte lipid envelope in the SC and play a crucial role in formation of an intact epidermal permeability barrier [23]. In the present study, the relationships between Cers and skin barrier function were determined by Pearson’s correlation analysis. Cer-EOS, Cer-NDS, and Cer-NS showed significant positive correlations with TEWL value. These findings may provide insights into the respective roles of Cers for the skin barrier function and warrant further investigation in studies on senile pruritus.

TAG was the only main lipid class that showed a significant decrease in senile pruritus. TAG is a minor component of lamellar bodies and the extracellular matrix of the SC in healthy skin [24, 25]. Reduction of TAG synthesis or increased degradation of TAG can lead to severe skin defects in humans and mice, and epidermal TAG metabolism plays a key role in maintenance of a proper permeability barrier in the skin [26, 27]. Previous studies showed that certain groups of TAG are correlated with impaired barrier function in AD [28]. Thus, TAG deficiency may be another contributing factor to the barrier dysfunction in senile pruritus.

Skin lipids not only play a structural role in maintaining the skin barrier, but also have a variety of biological and pathophysiological functions in the skin [29]. Sphingolipids, as one of the important pruritogenic substances, comprise a complex set of lipids including sphingomyelin and Cers. The activity of sphingomyelin deacylase, an enzyme that converts sphingomyelin into sphingosylphosphorylcholine (SPC) and free fatty acid, was elevated in AD patients [30]. Intradermal injection of SPC elicited hind-paw scratching in mice [31]. Although the pruritus in AD is orchestrated by the complex interplay of numerous different mediators, SPC was one of the contributing factors, suggesting that lipids may be an important pruritogenic substance in chronic pruritus [16]. In the present study, sphingomyelin and some Cers were positively correlated with pruritus score. Therefore, further investigations into the pruritogenic function of certain SSLs may be valuable.

### Study strengths and limitations

This study has several strengths. First, it is the first analysis of SSLs in senile pruritus to date, and allows characterization of the general lipid profiles as well as subgroup comparisons between patients and control subjects. Second, by using a new approach of independent data acquisition in LC-MS/MS, the sensitivity of the analysis was improved, which allowed comprehensive untargeted acquisition of molecular data. Third, Pearson product-moment correlation coefficient analysis was used to show the associations between SSLs and pruritus severity as well as between Cer profiles and TEWL value, thus providing a basis to identify the mechanism of lipid involvement in the skin barrier function and itching.

As one of the limitations of the study, the small sample size caused statistically insignificant differences in some measured lipids. Other factors such as patient dietary preferences, living habits, seasons, and geographical differences should also be taken into account. Therefore, studies on elderly patients with pruritus involving larger samples and more data are required to provide clearer evidence for the pathological changes in their lipid composition. Nevertheless, this study clearly demonstrates that SSLs are changed in senile pruritus. Future studies on certain SSL components toward novel therapies for senile pruritus could bring promising results.

## Conclusions

In summary, the study has presented a comprehensive quantitative characterization of SSLs and clearly revealed that senile pruritus patients have distinct SSL profiles in their SC compared with normal healthy controls. Differences in expression of 81 individual lipids were identified between the senile pruritus patients and control subjects. Correlation analyses showed that some SSL components were correlated with skin barrier damage. Although this study cannot demonstrate that the changes in SSLs cause the skin itching, the results indicate that certain SSLs may be the trigger or consequence of pruritus in elderly people. Therefore, further investigations on the pruritogenic function of certain SSLs may be valuable. Further research on senile pruritus is necessary to help elderly people find appropriate treatments for senile pruritus. Future studies focusing on certain SSL components may lead to novel therapies for senile pruritus.

## Supplementary information

**Additional file 1: Table 1.** Ameliorated Kawashima Itch Scale itch intensity scales.

**Additional file 2: Supplementary Table 2.** Lipid metabolites that significantly differ between elderly pruritus and control populations.

## Data Availability

All data generated or analysed during this study are available from the correspongding author on reasonable request.

## Abbreviations

ACN: Acetonitrile
IPA: Isopropanol
SSL: Skin surface lipids
TEWL: Transepidermal water loss
LC-MS/MS: Liquid chromatography coupled tandem mass spectrometry
Cers: Ceramides
DAGs: Diacylglycerols
FA: Fatty acids
PCs: Phosphatidylcholines
PE: Phosphatidylethanolaine
DGCC: Diacylceryl-3-O-carboxyhydroxymethylcholine
DGTS: Diacylglyceryl trimethylhomoserine
TAG: Triacylglycerol
SC: Stratum corneum
AD: Atopic dermatitis
SPC: Sphingosylphosphorylcholin
PLS-DA: Partial least squares discrimination analysis
