# Supplementary material for: Lipidomics profiling of skin surface lipids in senile pruritus
Source: Lipids Health Dis. 2020 Jul 16;19:171. doi: 10.1186/s12944-020-01347-y (PMC7364579; doi:10.1186/s12944-020-01347-y)
Supplement: Supplementary file 1 — Additional file 1: Table 1. Ameliorated Kawashima Itch Scale itch intensity scales. [file 12944_2020_1347_MOESM1_ESM.docx]

**Table 1 Ameliorated Kawashima Itch Scale itch intensity scales**

| Score | Pruritus state during the day | Pruritus state during the night |
| --- | --- | --- |
| 4 | Very severe, interfering with daily activities | Very severe, interfering with sleep |
| 3 | Severe, very annoying, substantially interfering with daily activities | Severe, very annoying, substantially interfering with sleep |
| 2 | Moderate, annoying and troublesome, may interfering with daily activities | Moderate, annoying and troublesome, may interfering with sleep |
| 1 | Mild, not annoying or troublesome | Mild, not annoying or interfering with sleep |
| 0 | None | None |
