# Supplementary material for: Lipidomics profiling of skin surface lipids in senile pruritus
Source: Lipids Health Dis. 2020 Jul 16;19:171. doi: 10.1186/s12944-020-01347-y (PMC7364579; doi:10.1186/s12944-020-01347-y)
Supplement: Supplementary file 2 — Additional file 2: Supplementary Table 2. Lipid metabolites that significantly differ between elderly pruritus and control populations. [file 12944_2020_1347_MOESM2_ESM.docx]

**Supplementary Table2: Lipid metabolites that significantly differ between elderly pruritus and control populations**

| **Class** | **Metabolite name** |  | **Formula** |  | ***P* value** |  | **level（control/patients）** |
| --- | --- | --- | --- | --- | --- | --- | --- |
| Cer | Cer t62:1 |  | C62H123NO5 | | 0.004 |  | **↑** |
| Cer | Cer t34:0 |  | C34H69NO5 |  | 0.045 |  | **↑** |
| Cer-AP | Cer-AP t48:0; Cer-AP t23:0/25:0 |  | C48H97NO5 |  | 0.044 |  | **↑** |
| Cer-BDS | Cer-BDS d41:1; Cer-BDS d25:0/16:1 |  | C41H81NO4 |  | 0.025 |  | **↑** |
| Cer-EOS | Cer-EOS d60:2; Cer-EOS d27:1/33:1 |  | C60H115NO5 | | 0.012 |  | **↑** |
| Cer-EOS | Cer-EOS d61:1 |  | C61H119NO5 | | 0.043 |  | **↑** |
| Cer-EOS | Cer-EOS d59:2; Cer-EOS d27:1/32:1 |  | C59H113NO5 | | 0.046 |  | **↑** |
| Cer-EOS | Cer-EOS d58:2; Cer-EOS d27:1/31:1 |  | C58H111NO5 | | 0.051 |  | **↑** |
| Cer-NDS | Cer-NDS d41:0; Cer-NDS d23:0/18:0 |  | C41H83NO3 |  | 0.003 |  | **↑** |
| Cer-NDS | Cer-NDS d38:0; Cer-NDS d20:0/18:0 |  | C38H77NO3 |  | 0.006 |  | **↑** |
| Cer-NDS | Cer-NDS d40:0; Cer-NDS d22:0/18:0 |  | C40H81NO3 |  | 0.006 |  | **↑** |
| Cer-NDS | Cer-NDS d38:2; Cer-NDS d19:0/19:2 |  | C38H73NO3 |  | 0.015 |  | **↑** |
| Cer-NDS | Cer-NDS d42:0; Cer-NDS d18:0/24:0 |  | C42H85NO3 |  | 0.016 |  | **↑** |
| Cer-NDS | Cer-NDS d40:2; Cer-NDS d19:0/21:2 |  | C40H77NO3 |  | 0.019 |  | **↑** |
| Cer-NDS | Cer-NDS d44:0; Cer-NDS d20:0/24:0 |  | C44H89NO3 |  | 0.030 |  | **↑** |
| Cer-NDS | Cer-NDS d36:2; Cer-NDS d19:0/17:2 |  | C36H69NO3 |  | 0.032 |  | **↑** |
| Cer-NDS | Cer-NDS d34:0; Cer-NDS d17:0/17:0 |  | C34H69NO3 |  | 0.039 |  | **↑** |
| Cer-NP | Cer-NP t46:0; Cer-NP t21:0/25:0 |  | C46H93NO4 |  | 0.023 |  | **↑** |
| Cer-NP | Cer-NP t47:0; Cer-NP t21:0/26:0 |  | C47H95NO4 |  | 0.052 |  | **↑** |
| Cer-NS | Cer-NS d44:1; Cer-NS d18:1/26:0 |  | C44H87NO3 |  | 0.001 |  | **↑** |
| Cer-NS | Cer-NS d46:1; Cer-NS d20:1/26:0 |  | C46H91NO3 |  | 0.002 |  | **↑** |
| Cer-NS | Cer-NS d40:1; Cer-NS d16:1/24:0 |  | C40H79NO3 |  | 0.002 |  | **↑** |
| Cer-NS | Cer-NS d42:1; Cer-NS d18:1/24:0 |  | C42H83NO3 |  | 0.002 |  | **↑** |
| Cer-NS | Cer-NS d50:1; Cer-NS d22:1/28:0 |  | C50H99NO3 |  | 0.003 |  | **↑** |
| Cer-NS | Cer-NS d34:1; Cer-NS d18:1/16:0 |  | C34H67NO3 |  | 0.004 |  | **↑** |
| Cer-NS | Cer-NS d41:1; Cer-NS d17:1/24:0 |  | C41H81NO3 |  | 0.004 |  | **↑** |
| Cer-NS | Cer-NS d48:1; Cer-NS d20:1/28:0 |  | C48H95NO3 |  | 0.005 |  | **↑** |
| Cer-NS | Cer-NS d36:1; Cer-NS d18:1/18:0 |  | C36H71NO3 |  | 0.007 |  | **↑** |
| Cer-NS | Cer-NS d58:3; Cer-NS d17:3/41:0 |  | C58H111NO3 | | 0.007 |  | **↑** |
| Cer-NS | Cer-NS d45:1; Cer-NS d20:1/25:0 |  | C45H89NO3 |  | 0.008 |  | **↑** |
| Cer-NS | Cer-NS d44:2; Cer-NS d20:1/24:1 |  | C44H85NO3 |  | 0.010 |  | **↑** |
| Cer-NS | Cer-NS d42:3; Cer-NS d18:2/24:1 |  | C42H79NO3 |  | 0.011 |  | **↑** |
| Cer-NS | Cer-NS d35:2; Cer-NS d18:1/17:1 |  | C35H67NO3 |  | 0.012 |  | **↑** |
| Cer-NS | Cer-NS d42:2; Cer-NS d18:1/24:1 |  | C42H81NO3 |  | 0.015 |  | **↑** |
| Cer-NS | Cer-NS d45:1; Cer-NS d19:1/26:0 |  | C45H89NO3 |  | 0.018 |  | **↑** |
| Cer-NS | Cer-NS d43:2; Cer-NS d17:1/26:1 |  | C43H83NO3 |  | 0.019 |  | **↑** |
| Cer-NS | Cer-NS d40:2; Cer-NS d16:1/24:1 |  | C40H77NO3 |  | 0.020 |  | **↑** |
| Cer-NS | Cer-NS d43:1; Cer-NS d17:1/26:0 |  | C43H85NO3 |  | 0.020 |  | **↑** |
| Cer-NS | Cer-NS d60:3; Cer-NS d18:1/42:2 |  | C60H115NO3 | | 0.021 |  | **↑** |
| Cer-NS | Cer-NS d36:2; Cer-NS d18:1/18:1 |  | C36H69NO3 |  | 0.021 |  | **↑** |
| Cer-NS | Cer-NS d61:3; Cer-NS d18:1/43:2 |  | C61H117NO3 | | 0.022 |  | **↑** |
| Cer-NS | Cer-NS d47:1; Cer-NS d21:1/26:0 |  | C47H93NO3 |  | 0.023 |  | **↑** |
| Cer-NS | Cer-NS d36:2; Cer-NS d20:1/16:1 |  | C36H69NO3 |  | 0.033 |  | **↑** |
| Cer-NS | Cer-NS d48:2; Cer-NS d22:1/26:1 |  | C48H93NO3 |  | 0.033 |  | **↑** |
| Cer-NS | Cer-NS d34:2; Cer-NS d18:2/16:0 |  | C34H65NO3 |  | 0.034 |  | **↑** |
| Cer-NS | Cer-NS d45:2; Cer-NS d21:1/24:1 |  | C45H87NO3 |  | 0.036 |  | **↑** |
| Cer-NS | Cer-NS d50:4; Cer-NS d20:1/30:3 |  | C50H93NO3 |  | 0.038 |  | **↑** |
| Cer-NS | Cer-NS d41:2; Cer-NS d17:1/24:1 |  | C41H79NO3 |  | 0.038 |  | **↑** |
| Cer-NS | Cer-NS d44:1; Cer-NS d18:1/26:0 |  | C44H87NO3 |  | 0.041 |  | **↑** |
| Cer-NS | Cer-NS d34:2; Cer-NS d18:1/16:1 |  | C34H65NO3 |  | 0.044 |  | **↑** |
| Cer-NS | Cer-NS d62:3; Cer-NS d18:1/44:2 |  | C62H119NO3 | | 0.045 |  | **↑** |
| Cer-NS | Cer-NS d42:2; Cer-NS d18:2/24:0 |  | C42H81NO3 |  | 0.054 |  | **↑** |
| Cer-OS | Cer-OS d46:1; Cer-OS d23:1/23:0 |  | C46H91NO4 |  | 0.040 |  | **↑** |
| DAG | DAG 28:2e; DAG 8:0e/20:2 |  | C31H58O4 |  | 0.016 |  | **↑** |
| DAG | DAG 32:1; DAG 16:0-16:1 |  | C35H66O5 |  | 0.017 |  | **↑** |
| DAG | DAG 31:2e; DAG 15:1e/16:1 |  | C34H64O4 |  | 0.022 |  | **↑** |
| DAG | DAG 33:1e; DAG 17:0e/16:1 |  | C36H70O4 |  | 0.036 |  | **↑** |
| DAG | DAG 24:5e; DAG 22:5e/2:0 |  | C27H44O4 |  | 0.042 |  | **↑** |
| DAG | DAG 31:0e; DAG 22:0e/9:0 |  | C34H68O4 |  | 0.044 |  | **↑** |
| DGCC | DGCC 36:2; DGCC 18:1-18:1 |  | C46H85NO8 |  | 0.029 |  | **↑** |
| DGCC | DGCC 34:1 |  | C44H83NO8 |  | 0.052 |  | **↑** |
| DGTS | DGTS 24:1 |  | C34H63NO7 |  | 0.019 |  | **↑** |
| DGTS | DGTS 27:1 |  | C37H69NO7 |  | 0.034 |  | **↑** |
| DGTS | DGTS 36:2; DGTS 18:1-18:1 |  | C46H85NO7 |  | 0.038 |  | **↑** |
| FA | FA 21:1 |  | C21H40O2 |  | 0.035 |  | **↑** |
| FA | FA 22:1 |  | C22H42O2 |  | 0.053 |  | **↑** |
| PC | PC 37:3 |  | C45H84NO8P | | 0.011 |  | **↑** |
| PC | PC 32:2 |  | C40H76NO8P | | 0.054 |  | **↑** |
| PE | PE 36:2; PE 18:1-18:1 |  | C41H78NO8P | | 0.040 |  | **↑** |
| Phytosphingosine | Phytosphingosine 19:0 |  | C19H41NO3 |  | 0.004 |  | **↑** |
| Phytosphingosine | Phytosphingosine 20:0 |  | C20H43NO3 |  | 0.047 |  | **↑** |
| Sphingosine | Sphingosine 18:1 |  | C18H37NO2 |  | 0.041 |  | **↑** |
| Sphingosine | Sphingosine 24:1 |  | C24H49NO2 |  | 0.052 |  | **↑** |
| TAG | TAG 40:2e; TAG 8:0e-16:1-16:1 |  | C43H80O5 |  | 0.005 |  | **↓** |
| TAG | TAG 60:13e; TAG 16:3e-22:5-22:5 |  | C63H98O5 |  | 0.019 |  | **↓** |
| TAG | TAG 62:17e; TAG 18:5e-22:6-22:6 |  | C65H94O5 |  | 0.021 |  | **↓** |
| TAG | TAG 60:8e; TAG 20:4e-20:2-20:2 |  | C63H108O5 |  | 0.031 |  | **↑** |
| TAG | TAG 54:3; TAG 18:1-18:1-18:1 |  | C57H104O6 |  | 0.034 |  | **↓** |
| TAG | TAG 56:10e; TAG 20:5e-18:2-18:3 |  | C59H96O5 |  | 0.043 |  | **↓** |
| TAG | TAG 54:3; TAG 18:0-18:1-18:2 |  | C57H104O6 |  | 0.045 |  | **↓** |
| TAG | TAG 58:8; TAG 18:1-18:1-22:6 |  | C61H102O6 |  | 0.046 |  | **↓** |
